# Resolution of long-term severe irritable bowel syndrome following fecal microbiota transplantation: A case report and microbiota analysis

**DOI:** 10.1080/29933935.2025.2487905

**Published:** 2025-04-20

**Authors:** Elena Montenegro-Borbolla, Jeanine Wakim El-Khoury, Claire Bertelli, Alain Schoepfer, Benoît Guery, Tatiana Galperine

**Affiliations:** a Infectious Diseases Service, Lausanne University Hospital and University of Lausanne, Lausanne, Switzerland; b Institute of Microbiology, Lausanne University Hospital and University of Lausanne, Lausanne, Switzerland; c Division of Gastroenterology and Hepatology, University Hospital Lausanne - CHUV, Lausanne, Switzerland; d French Group of Fecal Microbiota Transplantation (GFTF), Paris, France

**Keywords:** Irritable bowel syndrome, IBS-C, fecal microbiota transplantation (FMT), microbiota analysis, engraftment

## Abstract

The diagnosis and management of irritable bowel syndrome (IBS) is challenging due to its complex symptoms and inconsistent treatment responses. Given the important role of gut microbiota in gastrointestinal health, fecal microbiota transplantation (FMT) is a promising intervention. We describe the case of a 55-y-old woman without prior gastrointestinal issues who, following severe depression, developed multiple gastrointestinal symptoms, including abdominal pain, fluctuating bowel habits, and a persistent burning sensation in her mouth and upper gastrointestinal tract. At Lausanne University Hospital, she was diagnosed with IBS resistant to multiple lines of treatment and a multidisciplinary team proposed multiple oral FMTs. One-month post-FMT, her gastrointestinal symptoms significantly improved and remained better after a year, with only the burning sensation persisting. Analysis of pre- and post-FMT samples and donor material, using 16S rRNA amplicon metagenomics, revealed a 90% genus-level taxonomic overlap between the patient and the donor. The observed changes in the relative abundance of these genera, including the enrichment of beneficial gut commensals, as well as the elimination of IBS-associated genera likely supported her recovery. Overall, FMT led to substantial improvement in her long-standing gastrointestinal symptoms.

## Introduction

Irritable bowel syndrome (IBS) is a gut-brain axis disorder defined by the Rome IV criteria^12^ as recurrent abdominal pain at least 1 d per week in the last 3 months, along with two or more of the following criteria: pain related to defecation, a change in stool frequency, and/or a change in stool form. These criteria must have been met for the last 3 months, with symptom onset at least 6 months before diagnosis. IBS subtypes are defined by the predominant bowel habit (>25% of bowel movements) based on stool form on days with at least one abnormal bowel movement: IBS with predominant diarrhea (IBS-D), IBS with predominant constipation (IBS-C), IBS with mixed bowel habits (IBS-M), and IBS unclassified (IBS-U). First-line treatments include changes in diet and lifestyle, followed by pharmacological treatments to alleviate the symptoms, including antibiotics, anti-inflammatory drugs, and anti-depressants.^22^ In the past decade, fecal Microbiota Transplantation (FMT) has gained attention as a potential therapy for IBS.^22^ FMT is a medical treatment consisting in the transfer of the intestinal microbiota from a healthy donor to a recipient with the intent to modulate the recipient’s microbiome for therapeutic purposes. While FMT is currently established for treating recurrent *Clostridioides difficile* infections,^43^ its effectiveness in IBS remains controversial.^1,10,17,24^ In contrast, some clinical benefit has been observed in IBS patients treated with probiotics^22,25,48^ Herein, we describe the case of a 55-y-old woman who presented with longstanding gastrointestinal symptoms refractory to conventional as well as some experimental therapies (refractory IBS defined as at least three failed recommended IBS treatments) and attended the Infectious Diseases and the Gastroenterology Departments of Lausanne University Hospital (CHUV, Switzerland). She was diagnosed with **IBS-C** and was administered FMT, successfully improving her symptoms. We hereby aimed to investigate the dynamics of the intestinal microbiota by assessing microbial engraftment post-FMT and understanding changes that potentially contributed to her recovery. Although typically defined by the incorporation of new bacterial strains from the donor, we also considered engraftment as the shift in microbiota community composition and abundance to reflect that of the donor.

### Case presentation

We report on the case of a 55-y-old woman who presented to our clinic with no relevant familial or personal medical history. She never had gastrointestinal symptoms, and her bowel movements were normal, consisting of one Bristol IV stool per day until the end of 2019, when she presented an episode of severe depression with anxiety and insomnia. She was started on chlorprothixene, lorazepam, zolpidem, and mirtazapine in June 2020. A few days later, she presented severe acute cramping abdominal pain with a sensation of a “knot” in the epigastric region and the right flank without fever, vomiting or diarrhea. Clinical and biological evaluation were normal, and COVID-19 was ruled out. At the emergency department, the abdominal-pelvic CT-scanner showed a homogeneous density 4-cm diameter tissue mass in a jejunal loop associated with circumferentially thickened walls (up to 1 cm), lying just below the pancreatic head, extending under the body of the pancreas which had a normal appearance. A pancreatic tumor or a jejunal lymphoma was subsequently ruled out by Magnetic Resonance Imaging (MRI) and laparoscopy in July 2020 with the latter identifying an internal right para-duodenal hernia via the Treitz angle that was reduced with closure of the anterior para-duodenal window.

**Figure 1.**
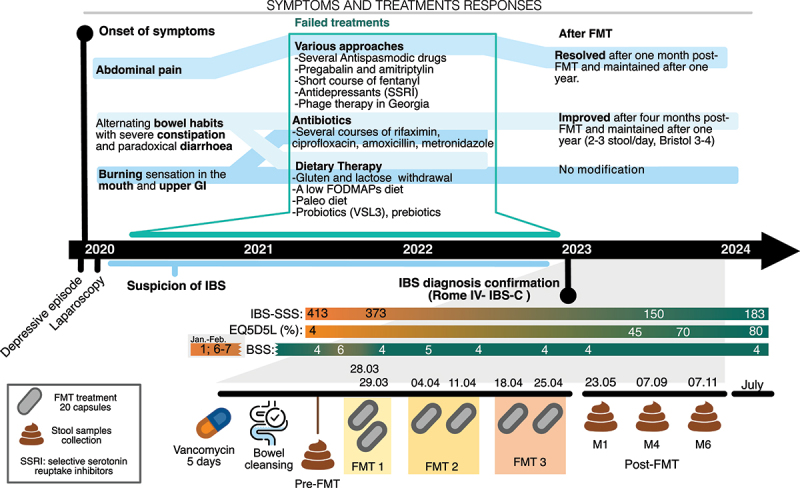
Timeline of symptoms, diagnosis, and treatment. The upper half illustrates the initial symptoms, unsuccessful treatments from 2020 to 2023, and symptom progression following FMT. Colored shading connects the onset symptoms with corresponding failed treatments and post-fmt outcomes. The lower half provides additional clinical details, with the gray-shaded area highlighting the 2023 timeline, including questionnaire scores, FMT treatments, and sample collection time points. IBS-SSS: irritable bowel syndrome severity scoring system; BSS: Bristol stool scale.

After the surgery, abdominal pain progressively increased and the patient developed bloating and alternating bowel habits with constipation and diarrhea consisting of Bristol VII stools, up to three times a day, and lost 2–4 kg of weight. She also had a generalized burning sensation spanning the whole abdomen, the retrosternal region, and the mouth. Until 2023, an extensive workup was performed (Table 1) in several hospital centers across Switzerland and concluded to IBS-C with overflow diarrhea as well as hypersensitive esophagus, both triggered by the severe episode of depression that she presented at the end of 2019. Many therapies were tried (Figure 1) but yielded no clinical response.

**Table 1.** List of investigations.

List of Investigations performed	Negative/Normal	Positive
**Blood tests**		
Celiac disease serology*	**x**	
Inflammatory markers (CRP, WBC)	**x**	
Anemia markers (Hb, MCV, ferritin)	**x**	
Serology for *Yersinia enterocolitica*	**x**	
Thyroid Stimulating Hormone (TSH)	**x**	
C1-esterase-inhibitor activity	**x**	
**Stool tests**		
Fecal calprotectin	**x**	
Stool cultures**		***C. jejuni, Blastocystis hominis^ᶧ^***
Stool Antigen for *H. pylori*		**x**^*ɤ*^
**Endoscopic tests**		
Gastroscopy	**x**^**¥**^	
Colonoscopy	**x**^¶^	
**Abdominal imaging**		
Abdominal-pelvic CT-scanner	**x**^¶^	
Abdominal-pancreatic MRI	**x**	
Gastric emptying scintigraphy	**x**	
**Laparoscopy**		**x**^**∫**^
**Anorectal & Esophageal functional tests**		
Anorectal high-definition manometry		
Esophageal high-resolution manometry	**x**	
24-h esophageal pH-impedance off PPI	**x**^**£**^	
**Colon transit times**	**x (28.8 h)**	
**Lactulose breath test**		**x**^**∫**^
**Urinary tests (catecholamine, porphyria)**	**x**	
**Gynecological tests**	**x**	

Abbreviations: CRP, C-Reactive Protein; WBC, White Blood Cell Count; Hb, Hemoglobin; MCV, Mean Corpuscular Volume; *Anti-Deaminated Gliadin Peptide IgG and IgA, anti-tissue transglutaminase IgG and IgA with dosage of IgA and IgG; **Stool cultures for pathogenic bacteria (*C. jejuni, Salmonella, Shigella…)*, protozoa *(Giardia lamblia, Entamoeba histolytica, Cryptosporidium spp)* and helminths. *^ᶧ^C. jejuni* PCR positive, antigen negative so considered a colonizer; positive *Blastocystis hominis* treated with metronidazole. ^*ɤ*^Treated with metronidazole, tetracycline, potassic bismuth, Proton Pump Inhibitor (PPI) (eradication confirmed by a negative breath test and gastric biopsies off PPI). ^¥^Normal except for a microfocus of complete intestinal metaplasia localized to the gastric antrum); ^¶^Normal except colonic coprostasis as well as a 4-cm tissue density in a jejunal loop associated with circumferentially thickened walls (up to 1 cm), lying just below the pancreatic head, extending under the body of the pancreas which had a normal appearance; ^**∫**^Laparoscopy showed an internal paraduodenal hernia without neoplasia; ^£^No gastroesophageal reflux disease, but hypersensitive esophagus; ^∫^single-peak of hydrogen at 60 minutes, decreasing at 90 minutes; high methane levels ≥20 ppm all through the test.

The IBS was classified as severe with an Irritable Bowel Syndrome-Severity Scoring System (IBS-SSS)^14^ of more than 300 points and the case was discussed in a multidisciplinary meeting. Due to the failure of standard therapies and the high impact of symptoms on the patient’s quality of life, we proposed to perform three experimental FMTs that were administered orally with donations sourced from the same donor. Four months after the FMT, the evaluation revealed significant clinical improvement in diarrhea and abdominal pain, marked by a decrease of over 175 points in the IBS-SSS score (Figure 1) resulting in a significant improvement in her quality of life shown by an increase of at least 40% in the quality-of-life score. This clinical amelioration persisted over time, as assessed after over 12 months of follow-up. The patient provided written informed consent.

## Materials and methods

### Fecal microbiota transplantation

The donor was recruited and screened according to the Swiss Agency for therapeutic products (Swissmedic) regulation based on the current version of the European Directorate for the Quality of Medicines & HealthCare (EDQM) at the CHUV FMT center. The donor was a healthy voluntary Caucasian male aged 32 y with no specific diet. He was a nonsmoker, not taking any medication, and had a BMI of 25.5 kg/m^2^. His donation had already been utilized to treat *C. difficile* infection in recipients with a high efficacy rate of 95%.

The treatment consisted of a filtered solution of donor fecal microbiota (50 g) homogenized with sterile saline (6 mL) and addition of glycerol as a microbial cryopreserving agent in capsules. The capsules were manufactured under Good Manufacturing Practice (GMP) conditions at the CHUV Pharmacy. The FMT capsules followed the formulation by reference^47^ and were stored at −80°C.^23^ One FMT treatment involves taking 20 oral capsules Capsugel® DRCaps, Lonza, Switzerland), representing about 16–20 g of gram of feces, within an hour on two separate days. FMT pre-treatment consisted of 5 d of antibiotic (vancomycin) and bowel cleansing (macrogol solution) the day before FMT.

### IBS assessment

Abdominal symptoms were assessed using the IBS Symptom Severity Score (IBS-SSS),^19^ and health-related quality of life was measured using the standardized tool EQ-5D-5 L (EuroQuol 5-Dimensions 5-Levels).

### Stool collection and microbiota characterization

To study the impact of FMT on the gut microbiota of our patient, stool samples were collected at four different time points: After the vancomycin antibiotic treatment (before FMT), as well as 1, 4, and 6 months after the FMT (Figure 1) following the IHM SOP 005 criteria (https://human-microbiome.org/index.php?id=Sop&num=005). The material of the FMT capsules was also analyzed to establish the reference of the microbiota administered. The patient’s samples were preserved in eNAT® (COPAN, Italy) whilst the FMT material was represented by filtered stool encapsulated with 80% glycerol (see FMT manufacturing section). DNA was extracted with MagNA Pure (Roche, Basel, Switzerland), and regions V3-V4 of the 16S rRNA gene were amplified for library preparation according to the protocol “16S Metagenomic Sequencing Library Preparation” (Part. #15044223 Rev. B, Illumina, San Diego CA, USA). Reads were sequenced on Illumina MiSeq, and processed by zAMP, an in-house pipeline based on DADA2^36^ using the EBioCloud database^5^ for taxonomical assignment, using the default filtering parameters. Statistical analysis and visualization were done in the R programming language.^41^ All samples obtained over 370’000 reads (Table S1) and amplicon sequence variants (ASVs) with only one count were removed (1238 retained ASVs and 4 discarded). Compositional analyses were performed on the filtered non-rarefied dataset since all samples reached their plateau on their rarefaction curves (Figure S1).

## Results and discussion

**Figure 2.**
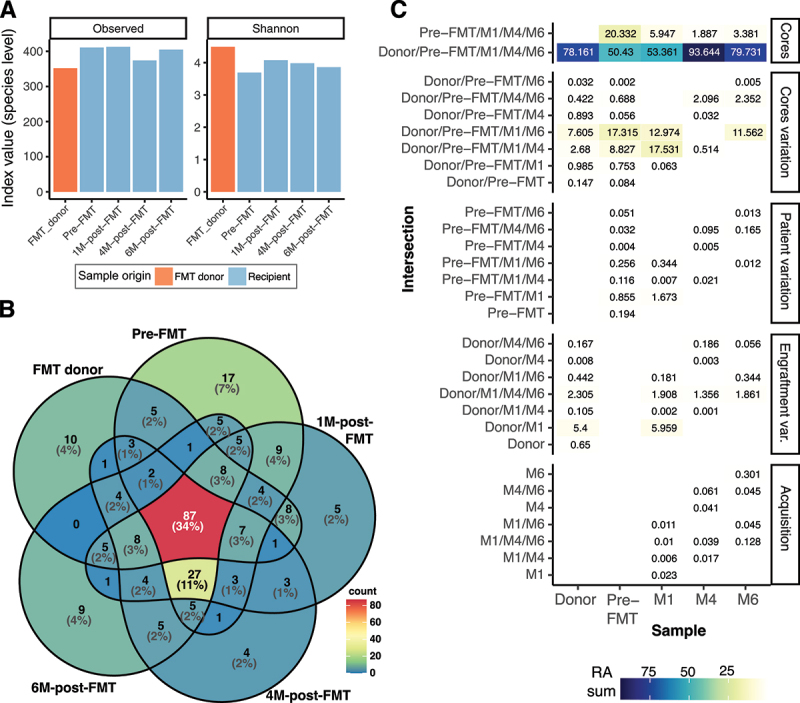
Bacterial abundance and distribution across samples. (A) Alpha diversity indexes at the ASV level of the different samples. Colours represent the origin of samples. (B) Venn diagram of genera shared among the various samples based on absence/presence. Percentages corresponding to 0% have been removed to ease the visualization. (C) Relative abundance (RA) of the genera in each intersection of samples. Categories represent: (core) shared and patient-specific genera; (core variation) genera shared between donor and patient, but missing in some patient samples post-fmt; (patient variation) genera from the patient-specific core that are missing in some of the samples post-fmt; (engraftment variation) bacteria absent before FMT in the patient and present in the donor, but that appear at different time points post-fmt; (acquisition) taxa absent pre-fmt and in the donor, or below detection limit, that may come from external sources, or was below limit of detection in earlier time-points.

Using 16S rRNA amplicon-based metagenomics, we analyzed the microbiota composition of patient’s fecal samples collected before FMT and 1, 4, and 6 months post-FMT, as well as the composition of the donor stool administered as capsules. Alpha diversity metrics revealed higher species richness in patient samples as compared to the FMT donor, but lower evenness (Figure 2A). Approximately one-third of the overall diversity at the genus level was found to be shared among all samples (Figure 2B), constituting the shared core genera that represented from 50% to 94% of the microbial communities within each sample (Figure 2C). When including the varying core genera (i.e. genera shared by both individuals but missing at some post-FMT time points, Figure 2C), the relative abundance of shared taxa amounts to over 90%, indicating a cohesive community between the donor and the recipient. Notably, the patient’s core microbiota, not shared with the donor, constituted 20% of the bacterial composition pre-FMT, diminishing to less than 3% 6 months post-FMT. This reduction suggests a gradual engraftment of the donor microbiota and replacement of the patient’s original microbiota. Other intersections detailed in Figure 2C refer to variations of patient’s unique genera, engrafted genera, and genera acquired from the environment, or below detection limit at other timepoints. They mainly consist of rare taxa, defined as those with a relative abundance below 1%. This finding is consistent with another longitudinal study of normal gut microbiota, which showed that while the abundance of dominant species remains relatively stable, rare taxa are more fluctuant and niche specific.^32^ Additionally, the microbiota composition of this IBS-C patient does not fully align with the genera composition reported in a previous study examining this topic (Table S2). Therefore, we will closely analyze shifts in genera relative abundance across the identified taxonomic intersections and their potential impact on the patient’s symptom remission. Overall, most of the bacterial composition at genus level was shared between the patient and donor, and this proportion even increased 4 months after FMT.

**Figure 3.**
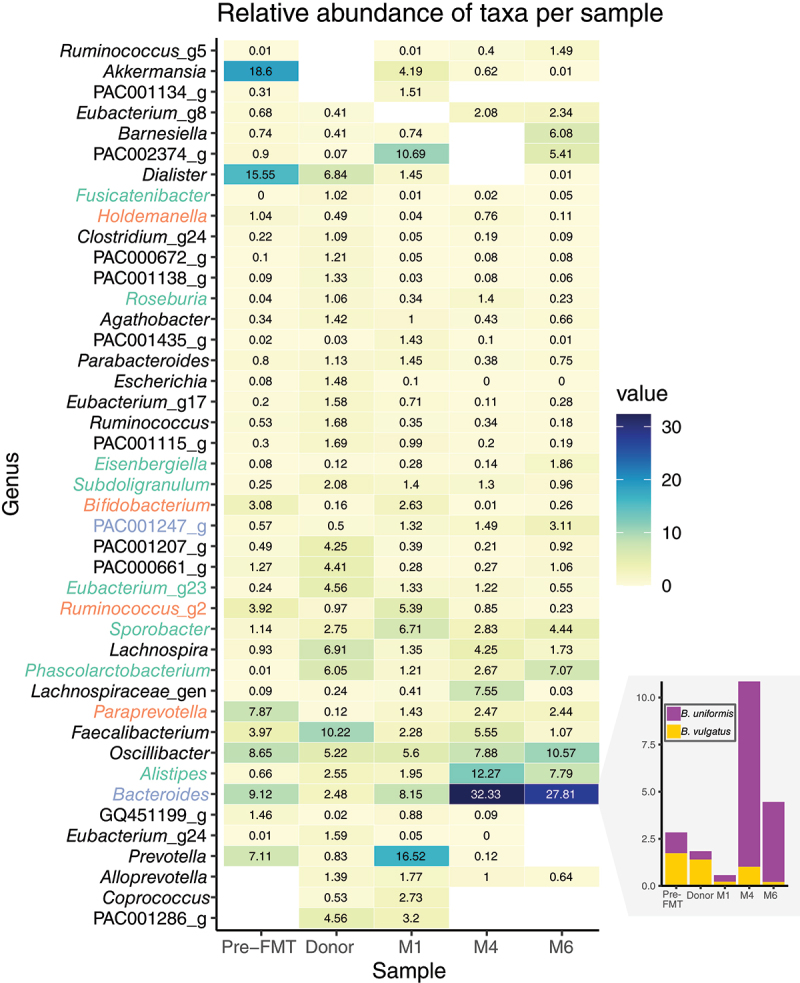
Relative abundance (%) of genera per sample. Only genera with a relative abundance > 1% in any sample are represented. Genera of the shared core taxa (present in all samples) are color-coded if there were boosted by FMT (green), increased after FMT (blue), or reduced by FMT (orange) by at least a two-fold change from pre-fmt to M6 post-fmt. Names in black remained stable. Bottom-right bar-plot represents the relative abundance (%) per sample of two Bacteroides species defined as “gatekeepers” by reference.^37^

The shared core genera can be categorized into four groups based on their relative abundance (RA) in each sample: (i) boosted by FMT, denoting genera with higher RA in the donor that experienced an increase following the patient’s recovery; (ii) increased after FMT, comprising genera already present in the patient which showed further increase after recovery; (iii) reduced by FMT, indicating a reduction in RA possibly due to the low abundance of these genera in the donor; and (iv) genera maintaining stability in the patient despite FMT (Table 2). Genera boosted by FMT include *Alistipes*, *Phascolarctobacterium*, *Lachnospira*, and *Sporobacter* (Figure 3). *Alistipes* spp. are commonly found in healthy gut microbiota, serving as potential short-chain fatty acid (SCFA) producers. Their roles were deemed protective in conditions such as colitis, yet potentially pathogenic in disorders like anxiety and depression.^34^ The *Lachnospira* genus, belonging to the SCFA-producing families, is strongly linked to vegetable-based diets,^42^ although its impact on IBS remains ambiguous.^31,45,49^ SCFAs participate in various gut homeostasis processes, including maintaining the mucosal barrier integrity and immune system regulation,^2^ therefore an increase in SCFA production could improve gut health status. Another key metabolite is GABA, which influences the gut-brain axis, and when modified into GABA-peptidoglycans by the microbiota, may act as a neuromodulator to alleviate visceral pain in IBS.^35^ *Sporobacter* genus has been shown to increase in children with IBS in response to a low fermentable substrate diet.^8^ Notably, the most abundant genus of post-symptom remission is *Bacteroides*, whose role remains controversial. It is among the most prevalent gut commensals,^4^ and certain species have been associated with successful FMT engraftment.^1,37^ Additionally, *Bacteroides sp*. strains from donor and recipient tend to coexist,^37^ potentially explaining the increase in this genus RA. *B. uniformis* and *B. vulgatus* have been suggested as “gatekeepers” of the recipient’s microbiota^37^ and their highest RA at 4 months post-FMT (Figure 3) coincides with the highest RA percentage of the patient’s core microbiota (Figure 2C). Consequently, the genera with an augmented RA after FMT may be considered beneficial taxa in our patient.

**Table 2.** Category of the shared core taxa and their logical definition.

Category	Conditions
Boosted by FMT	(Donor > Pre-FMT) &; (M6 > Pre-FMT)
Increased after FMT	(Pre-FMT > Donor) &; (M6 > Pre-FMT)
Reduced by FMT	(Pre-FMT > Donor) &; (Pre-FMT > M6)
Stable	Remaining taxa

The patient’s core microbiota comprises numerous rare taxa except for a few genera (Figure 3). Notably, *Akkermansia* spp. is of particular interest due to its recognized role as a probiotic in alleviating IBS symptoms.^10,15,18,30^ The genus constituted nearly 20% of the pre-FMT microbiota but declined to below 1% 4 months post-FMT (Figure 3). This reduction in *Akkermansia* spp. aligns with the typical abundance of *Akkermansia muciniphila* in healthy individuals, which ranges between 1% and 5%.^11,15^ While *A. muciniphila* is considered a beneficial bacterium, its elevated abundance has been associated with conditions such as gut inflammation and irritable bowel disease (IBD), where it contributes to mucosal barrier dysregulation.^6^ Therefore, the post-FMT reduction in *Akkermansia* relative abundance may have played a role in symptom alleviation. Additionally, *Ruminococcus_g5* initially present in low relative abundance before FMT, exhibited a two-order-of-magnitude increase post-FMT. However, another *Ruminococcus* genotyope (*Ruminococcus_g2*), also part of the shared microbiota, decreased following FMT (Figure 3). While *Ruminococcus* spp. has been identified as a commensal in disease-free guts,^32^ a study has associated it with IBS.^28^ Given the controversial findings across studies and reference databases, distinguishing between potential pathogenic, neutral commensal resident, and beneficial symbiotic strains remains challenging. Thus, despite their smaller abundance, the patient’s microbiota may have harbored beneficial bacteria absent from the donor’s that were increased after FMT as a result of changes in the community equilibrium.

Although absent at 4 months post-FMT, *Dialister* genus RA has substantially decreased similarly to *Akkermansia* (Figure 3). *Dialister* and *Phascolarctobacterium* occupy a distinct niche in succinate consumption, leading to the production of propionate as an energy source, thereby delineating the “succinotypes” within a microbiome.^3^ Specifically, the P-succinotype was proposed to indicate a healthy gut, while D-types may be associated with inflammation due to slower succinate consumption and its subsequent accumulation in the gut.^3^ Succinate accumulation in the gut activates the SUCNR1 receptor, stimulating pro-inflammatory response in macrophages, and has been reported in IBD microbiome dysbiosis.^21^ These succinotypes were shown to be mutually exclusive,^3^ aligning with our findings (Figure 3). Prior to FMT, the patient exhibited a high abundance of *Dialister* (15.55%) and low *Phascolarctobacterium* (0.01%), but the profile was reversed 6 months post-FMT (0.01% and 7.07%, respectively). We hypothesize that this shift in succinotype could have contributed to the patient’s recovery.

Beyond the shared and patient-specific core microbiota, the remaining intersections comprised a reduced number of rare taxa, except for *Dialister*, PAC002374_g (an unclassified *Bacteroidales*), and *Prevotella*. The latter has been identified as a biomarker of IBS-D and was strongly correlated to the severity of abdominal pain and discomfort as well as bloating. Curiously, *Prevotella* comprised nearly one-fifth of the patient’s RA one-month post-FMT, coinciding with the alleviation of abdominal pain, but not the stabilization of bowel habits (Figure 1). The presence of *Prevotella* has been associated with loose stools,^44^ a condition observed following FMT pretreatment. By 4 months post-FMT, the bowel habits were stable, and *Prevotella* had almost disappeared from the patient’s microbiota. *Bacteroides* and *Prevotella* are typically negatively correlated,^9^ consequently these changes post-FMT could have contributed to the bowel habits in this patient.

Only three new non-rare taxa were successfully engrafted onto the patient following FMT. One of these, *Alloprevotella*, was concerning, as it has been consistently associated with IBS across multiple studies.^13,26,40^ Fortunately, its RA diminishes below 1% at 6 months post-FMT (Figure 3). Among the engrafted taxa, *Coprococcus*, a SCFA producer,^20^ has been linked to healthy subjects compared to IBS patients.^27^ Despite its low relative abundance, *Coprococcus* may still contribute to a positive trajectory toward recovery post-FMT. Finally, factors beyond our taxonomical resolution (namely bacterial species and strains) or outside of the scope of this analysis (including bacterial functions, eukaryotes, metabolites, or viruses; patient’s immune status and diet) may also have played a role in the alleviation of symptoms after FMT.^7^

The success of engraftment can be assessed by the integration of the donor’s strains^1^ and depends upon various factors including the underlying pathology (infectious versus noninfectious) and the mode of administration, as well as prior antibiotic exposure preceding FMT.^24^ In our study, the patient underwent vancomycin antibiotic therapy, bowel cleansing, received multiple FMT doses via capsules, and exhibited gut inflammation, collectively diminishing microbial colonization resistance. Despite the limited resolution of 16S rRNA amplicon metagenomics, we observe considerable overlap in genera between the donor and the patient. We postulate that the replacement of strains from common genera may have contributed to this success^1,24^

However, strains acquired from the environment accounted on average 11.5% among the post-FMT bacterial landscape,^1^ suggesting the potential incorporation of exogenous strains beyond our direct investigation. Furthermore, our study’s scope is limited by its focus solely on bacterial composition, precluding evaluation of the influence of bacteriophage or metabolites on patient recovery.^33^ Additionally, our observations are biased by the vancomycin treatment administered to the IBS-C patient in preparation for FMT. Nevertheless, the remission of gastrointestinal symptoms 1 y after FMT treatment could indicate a positive impact of the new gut microbiota composition.

The use of FMT for IBS remains a topic of ongoing debate. The variability in therapeutic approaches for microbiota restoration, differences in FMT protocols (pre-treatment, donor and patient selection, route of administration, regimen, frequency, etc.), and limited study sample sizes make it challenging to draw definitive conclusions.^17,46^ While a few major randomized clinical trials incorporate bowel cleansing, antibiotic pretreatment to deplete the recipient’s microbiota is not a common practice.^16,17,29^ Singh and collaborators (2022) reported lower FMT engraftment in IBS-D patients pre-treated with antibiotics;^38,39^ however, their assessment was based on the incorporation of new donor OTUs in the recipients. As demonstrated in this study, symptom resolution in IBS-C may also result from shifts in relative abundance of pre-established genera. Evaluating the effects of FMT at the bacterial strain level requires shotgun metagenomics, and as mentioned earlier, the role of specific strains remains a subject of debate. Therefore, this case report highlights the need to reconsider FMT protocols for IBS-C and to analyze the effects of microbiota composition and function on patient outcomes with multi-omics approaches.

## Conclusion

In this study, a patient diagnosed with IBS-C exhibited successful alleviation of symptoms following FMT treatment. Analysis of the microbiota landscape pre- and post-transplant indicated that most of the bacterial composition at genus level is shared by the patient and donor. Changes in the relative composition of the microbiota or specific members at strain level, potentially contributing to remission of symptoms, included modifications of the succinotype, reduction in the relative abundance of IBS-associated genera, and increase in the commensal gut bacterial populations. Further larger scale studies should be undertaken to elucidate the importance of changes in microbial community function, alongside composition, and the potential role of other classes of microorganisms (viruses and in particular bacteriophages, fungi or protists). Nevertheless, our findings underscore the potential role of FMT in reshaping the gut microbiota composition and ameliorating symptoms in selected IBS-C patients.

## Supplementary Material

Supplemental Material

## Data Availability

The data for this study have been deposited in the European Nucleotide Archive (ENA) at EMBL-EBI under the accession number PRJEB81207. The R code and necessary files to replicate our results have been deposited on Zenodo (Montenegro-Borbolla, E. (2025). Dataset for Resolution of Long-Term Severe Irritable Bowel Syndrome Following Fecal Microbiota Transplantation: A Case Report and Microbiota Analysis. Zenodo.).
