# Supplementary material for: Resolution of long-term severe irritable bowel syndrome following fecal microbiota transplantation: A case report and microbiota analysis
Source: Gut Microbes Rep. 2025 Apr 20;2(1):2487905. doi: 10.1080/29933935.2025.2487905 (PMC12940158; doi:10.1080/29933935.2025.2487905)
Supplement: Supplemental Material [file KGMR_A_2487905_SM0325.docx]

SUPPLEMENTARY MATERIAL

**Table S1:** Read counts per sample

|  | Total reads | Maintained reads | Reads processing | Percentage of maintained reads |
| --- | --- | --- | --- | --- |
| Donor | 374’829 | 295’772 | 79’057 | 0.79 |
| Pre_FMT | 419’679 | 360’157 | 59’522 | 0.86 |
| M1 | 569’740 | 511’309 | 58’431 | 0.9 |
| M4 | 485’532 | 428’761 | 56’771 | 0.88 |
| M6 | 603’575 | 507’657 | 95’918 | 0.84 |

**Table S2**: Genera associated with IBS-C and IBS-C control from reference (50) , their relative abundance in the FMT donor and recipient in this study, and the potential impact of their relative abundance changes following FMT as per **Figure 3**..

| Phenotype | Genus | RA | FMT effect |
| --- | --- | --- | --- |
| IBS-C | *Parabacteroides* | ~1% | Unaffected |
|  | *Oscillibacter* | ~5-10% | Unaffected |
|  | *Eisenbergiella* | <2% | Increased |
|  | *Ruminiclostridum* | - | Not present |
|  | *Intestinimonas* | - | Not present |
|  | *Frisingicoccus* | - | Not present |
|  | *Hungatella* | - | Not present |
|  | *Negativibacilus* | - | Not present |
|  | *Dielma* | - | Not present |
|  | *Oscillospira* | - | Not present |
|  | *Christensenella* | - | Not present |
|  | *Barnesiella* | 0.7-6% | Increased at M6, but not by FMT |
|  | *Flavonibactor* | - |  |
|  | *Escherichia* | <1% patient  1.5% donor |  |
| IBS-C control | *Anaerosporobacter* |  |  |
|  | *Corynebacterium* |  |  |
|  | *Bulyricicoccus* |  |  |
|  | *Eubacterium eligens* group | <1% | Various groups of *Eubacterium* are present and one is increased after FMT |
|  | *Succinivirbrio* | - |  |
|  | *Roseburia* | ~1% | Increased after FMT |

**Figure S1**: Rarefaction curves. Vertical lines represent 150’000 (red) and 250’000 (blue) reads. “M1” and “M6” labels have been moved from the original plot for visualization clarity.
